# Supplementary material for: Autophagic cell death is dependent on lysosomal membrane permeability through Bax and Bak
Source: eLife. 2017 Nov 17;6:e30543. doi: 10.7554/eLife.30543 (PMC5697932; doi:10.7554/eLife.30543)
Supplement: Figure 1—source data 1. [file elife-30543-fig1-data1.pptx]

## Slide 1
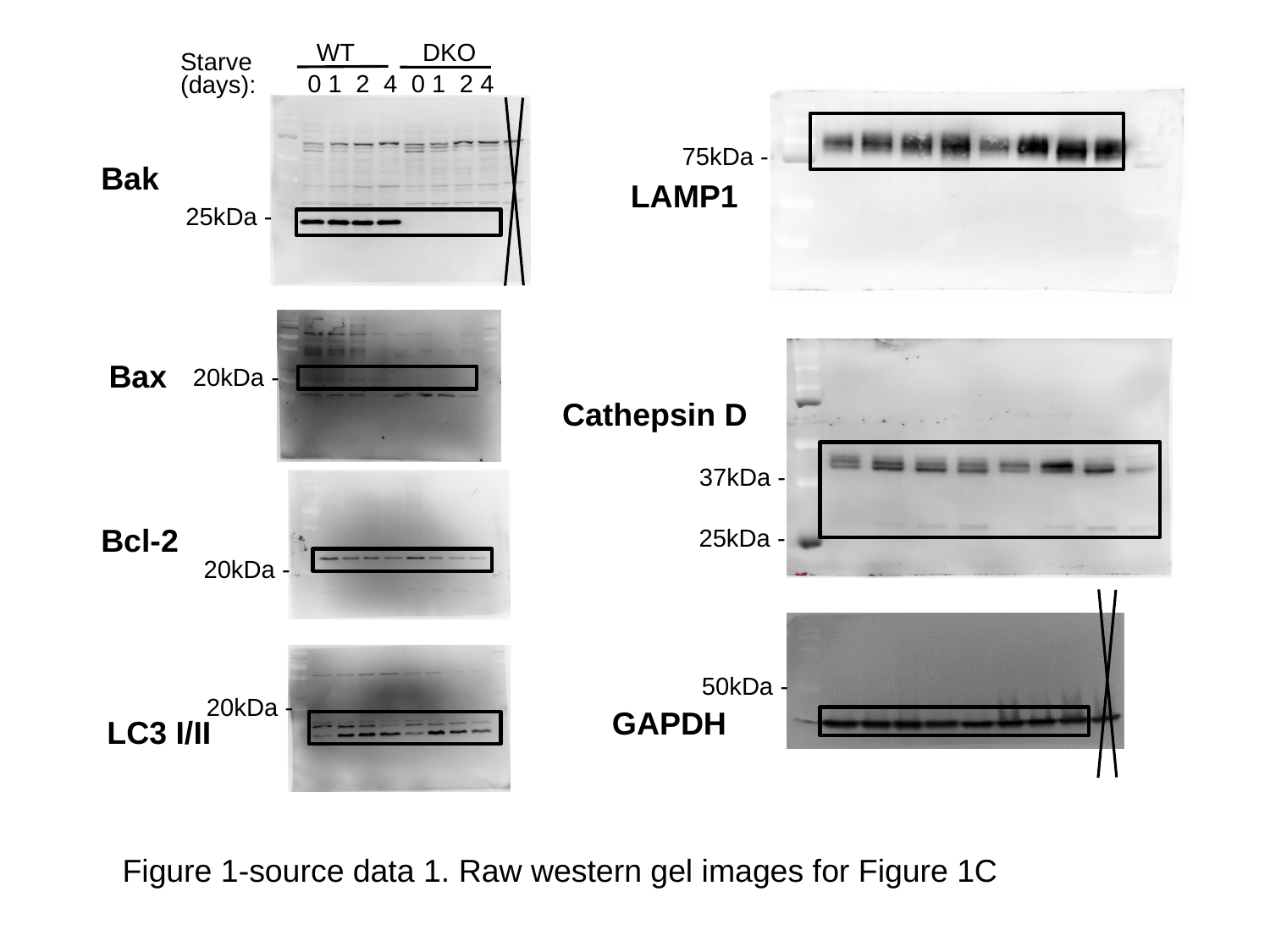

DKO
WT
Starve
(days):
0 1 2 4 0 1 2 4
75kDa -
Bak
LAMP1
25kDa -
Bax
20kDa -
Cathepsin D
37kDa -
Bcl-2
25kDa -
20kDa -
50kDa -
20kDa -
GAPDH
LC3 I/II
Figure 1-source data 1. Raw western gel images for Figure 1C
