# Supplementary material for: Autophagic cell death is dependent on lysosomal membrane permeability through Bax and Bak
Source: eLife. 2017 Nov 17;6:e30543. doi: 10.7554/eLife.30543 (PMC5697932; doi:10.7554/eLife.30543)
Supplement: Figure 2—figure supplement 1—source data 1. [file elife-30543-fig2-figsupp1-data1.pptx]

## Slide 1
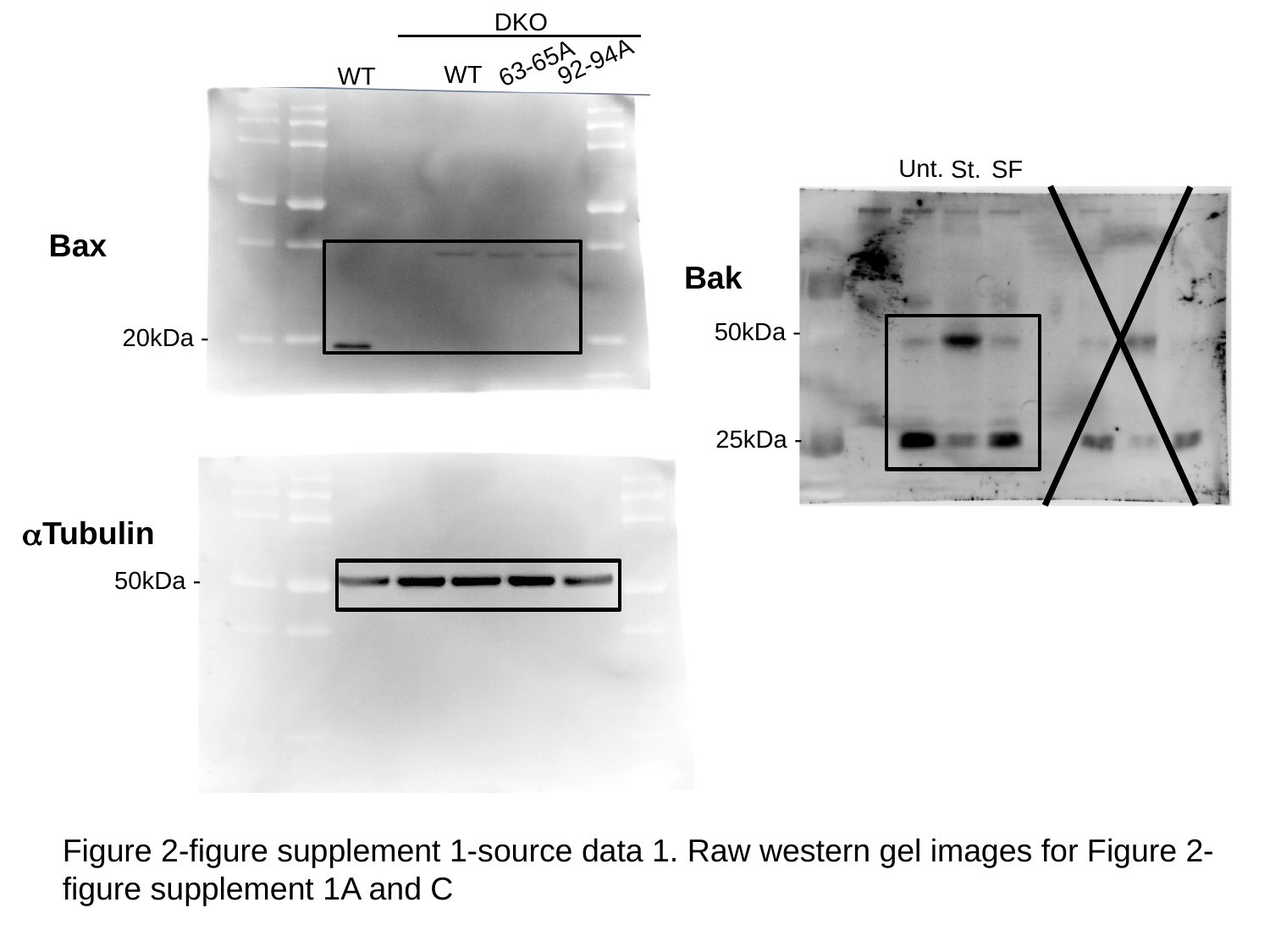

DKO
63-65A
92-94A
WT
WT
Unt.
St.
SF
50kDa -
25kDa -
Bax
Bak
20kDa -
aTubulin
50kDa -
Figure 2-figure supplement 1-source data 1. Raw western gel images for Figure 2-figure supplement 1A and C
