# Supplementary material for: Autophagic cell death is dependent on lysosomal membrane permeability through Bax and Bak
Source: eLife. 2017 Nov 17;6:e30543. doi: 10.7554/eLife.30543 (PMC5697932; doi:10.7554/eLife.30543)
Supplement: Figure 4—source data 1. [file elife-30543-fig4-data1.pptx]

## Slide 1
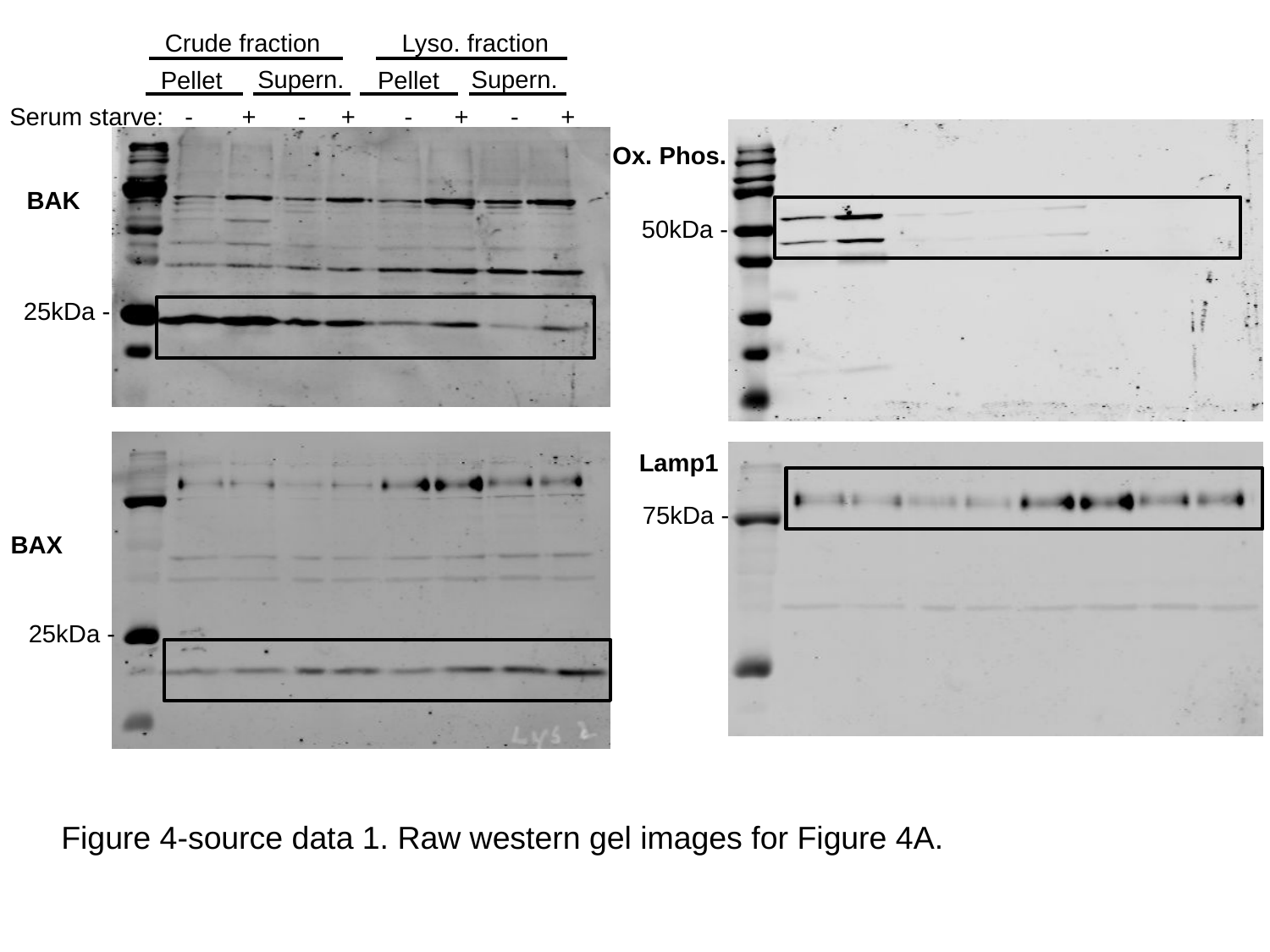

Crude fraction
Lyso. fraction
Supern.
Supern.
Pellet
Pellet
 Serum starve: - + - + - + - +
Ox. Phos.
BAK
50kDa -
25kDa -
Lamp1
75kDa -
BAX
25kDa -
Figure 4-source data 1. Raw western gel images for Figure 4A.
